# Computer use and stress, sleep disturbances, and symptoms of depression among young adults – a prospective cohort study

**DOI:** 10.1186/1471-244X-12-176

**Published:** 2012-10-22

**Authors:** Sara Thomée, Annika Härenstam, Mats Hagberg

**Affiliations:** 1 Occupational and Environmental Medicine, Department of Public Health and Community Medicine, University of Gothenburg, Gothenburg, Sweden; 2 Department of Work Science, University of Gothenburg, Gothenburg, Sweden

## Abstract

**Background:**

We have previously studied prospective associations between computer use and mental health symptoms in a selected young adult population. The purpose of this study was to investigate if high computer use is a prospective risk factor for developing mental health symptoms in a population-based sample of young adults.

**Methods:**

The study group was a cohort of young adults (n = 4163), 20–24 years old, who responded to a questionnaire at baseline and 1-year follow-up. Exposure variables included time spent on computer use (CU) in general, email/chat use, computer gaming, CU without breaks, and CU at night causing lost sleep. Mental health outcomes included perceived stress, sleep disturbances, symptoms of depression, and reduced performance due to stress, depressed mood, or tiredness. Prevalence ratios (PRs) were calculated for prospective associations between exposure variables at baseline and mental health outcomes (new cases) at 1-year follow-up for the men and women separately.

**Results:**

Both high and medium computer use compared to low computer use at baseline were associated with sleep disturbances in the men at follow-up. High email/chat use was negatively associated with perceived stress, but positively associated with reported sleep disturbances for the men. For the women, high email/chat use was (positively) associated with several mental health outcomes, while medium computer gaming was associated with symptoms of depression, and CU without breaks with most mental health outcomes. CU causing lost sleep was associated with mental health outcomes for both men and women.

**Conclusions:**

Time spent on general computer use was prospectively associated with sleep disturbances and reduced performance for the men. For the women, using the computer without breaks was a risk factor for several mental health outcomes. Some associations were enhanced in interaction with mobile phone use. Using the computer at night and consequently losing sleep was associated with most mental health outcomes for both men and women. Further studies should focus on mechanisms relating information and communication technology (ICT) use to sleep disturbances.

## Background

The widespread use of modern information and communication technology (ICT) in work life and private life follows in the wake of rapid advances in technology and popularization of different devices and applications, implying fast changes in exposure profiles in the population over the past few decades. The issue of possible negative effects of exposure to ICT has been raised by various groups. Musculoskeletal symptoms and ergonomics in relation to computer use and different input devices have been examined [1-3], but also, mental health effects have been considered (e.g. [4]). The term techno-stress emerged more than two decades ago, to describe stress reactions in relation to computer use [5]. It was suggested that computer use can lead to psychophysiological stress reactions due to occupational strain, and that these reactions can become conditioned to the computer work environment, leading to symptoms associated with computer use [5,6]. The term ICT stress has been used to describe stress induced by interruptions at work, time pressure, and technical problems in connection with ICT use [7].

In 2010, 91% of the Swedish population (16–74 years old) had access to the Internet at home [8]. Young adults, 20–24 years old, were the most frequent users compared to all other age groups [9]. In a previous prospective cohort study, we found associations between high use of ICT, including chatting, emailing, Internet surfing, the sum of hours spent at the computer and mobile phone per week, and the number of mobile phone calls and text messages (SMSs) per day, and reported mental health symptoms among college and university students aged 19–25 years [10]. The study was followed by a qualitative interview study with 32 high users of ICT who had reported mental health symptoms at 1-year follow-up in the cohort questionnaire [11]. Based on these young adults’ own concepts and ideas, a model of possible paths for associations between ICT use and mental symptoms was proposed via consequences of high quantitative computer or mobile phone use, negative qualitative use, and user problems. Consequences of computer use included spending more time than planned at the computer (e.g., working, gaming, or chatting), leading to time pressure, neglect of other activities and personal needs (e.g., breaks, physical activity, social interaction, sleep), exposure to bad ergonomics, and mental overload. Chatting or emailing interrupted other tasks, with difficulties filtering important from unimportant messages, leading to mental overload. Getting stuck in what was perceived as unproductive activities, such as game playing, or “gaming,” was another concern, and participants could also relate to having insufficient or dislocated sleep after sitting up late in front of the computer because of getting stuck in tasks, meeting deadlines, chatting, or gaming. In a study among Finnish adolescents [12], intensive computer use among the boys was a risk for poor perceived health through deteriorated sleeping habits and waking time tiredness. (For the girls, it was intensive mobile phone use that was directly associated with poor perceived health, likewise through deteriorated sleeping habits and waking time tiredness).

There has been a growing number of publications concerning ICT addiction [13]. Internet addiction has been proposed as a specific psychiatric illness [14]; however, this form of problematic Internet use could also be considered to share elements with impulse control disorders and be related to the specific activities done on the Internet (such as gambling) rather than the Internet per se [15,16]. Internet addiction has been associated with sleep disorders and depression among adults [17] and adolescents [18]. Most studies we have found concerning problematic Internet use and mental health have a cross-sectional design, but one exception is a prospective study among Chinese adolescents 13–18 years old [19], where Internet addicts, compared to non-pathological users, had a relative risk of about 2.5 for new cases of depression (using the Zung Depression Scale).

Another focus has been computer gaming. Playing computer games is more common among men and boys than among women or girls (e.g. [12,20,21]). In a longitudinal study among youths, pathological gaming predicted higher levels of depression, anxiety, social phobia, and poor school performance [22]. It seemed to be a long-term exposure, as most (84%) of the youths who were pathological gamers at baseline were still pathological gamers after 2 years.

Furthermore, in our interview study [11], social isolation was a concern. A negative loop was suggested, where people who are already lonely may have a preference for using computers, which in turn could increase their tendency to lack real-life contacts, and lead to an even higher use. This is in line with the findings of Morahan-Martin and Schumacher [23]. Positive effects of computer use were also listed, such as efficiency, access to information, fun and recreation, the ease of keeping up social contacts, and access to social support [11]. We concluded that there seemed to be many factors in different domains that should be taken into consideration in epidemiological studies concerning associations between ICT use and mental symptoms.

While we have previously performed a prospective study on computer use in relation to mental health symptoms in a selected university student cohort [10], it is important to examine possible prospective associations between computer use and mental health outcomes in a more general and heterogeneous population of young adults. The present study was performed in a population-based sample of young adults. We have recently performed a study on mobile phone use and mental health outcomes in the same cohort population [24]. Frequent mobile phone use was a prospective risk factor for sleep disturbances in the men, and for symptoms of depression in the men and women.

### Aims

The purpose of this study was to investigate if high computer use is a risk factor for developing mental health symptoms in a population sample of young adults. Specific aims included examining associations, if any, between time spent on computer use in general, time spent on communication (emailing or chatting) in leisure, time spent on computer gaming, using the computer without breaks, and getting stuck behind the computer screen at night and thus losing sleep, on the one hand, and perceived stress, sleep disturbances, and symptoms of depression, on the other. Furthermore, we wanted to explore perceived social support in relation to computer use. As a final goal, we aimed to examine potential interaction between computer and mobile phone use on mental health symptoms.

## Methods

### Study population and data collection

**Figure 1:**
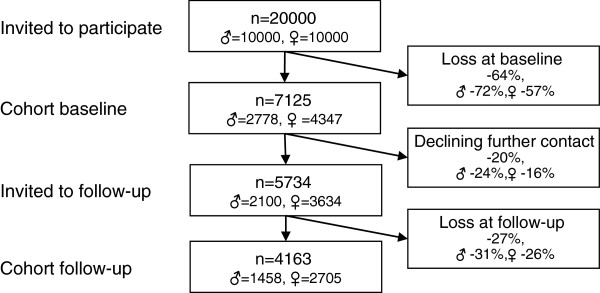
**Study population.** The participation process from study population to study group.

The study population consisted of a cohort of young adults (Figure 1), 20–24 years old (age span corresponding to the United Nations’ definition of young adults [25]). A cohort of 10000 men and 10000 women, born between 1983 and 1987, were randomly selected from the general population from a registry held by theSwedish Tax Agency, 50% living in the County of Västra Götaland, and 50% in the rest of Sweden. In October 2007, a questionnaire [26] containing questions about health, work- and leisure-related exposure factors, demographic factors, and psychosocial factors was sent to the selected young adults by post. Besides returning the postal questionnaire it was also possible to complete the questionnaire online if desired. As an incentive to respond, a lottery ticket (value approx. 1 Euro) was attached to the cover letter and could be used regardless of participation in the study. Two reminders were sent by post. The response rate at baseline was 36% (n = 7125; 2778 men and 4347 women). Twelve months later, those respondents who had indicated that they would consider participating in future studies (n = 5734) were invited to respond to an identical questionnaire, this time administered via the Web. The data collection process was similar to that at baseline, with the addition of a third reminder offering a paper version of the questionnaire and two cinema tickets for participating. The response rate at follow-up was 73% (Figure 1; n = 4163; 1458 men and 2705 women). The study was approved by the Regional Ethics Review Board in Gothenburg, Sweden (Reg. no. 191–05 and 876–11).

### Exposure variables

Information about computer use was collected from the cohort study questionnaire at baseline, including average time spent daily on general computer use, on emailing or chatting in leisure, and on computer gaming, as well as how often the computer was used for more than 2 hours without breaks, and how often sleep was lost because of getting stuck late at night by the computer. Also, information about mobile phone use (average number of calls and SMSs sent and received per day) was collected from the questionnaire. Response data were categorical, and were further divided into *high*, *medium*, and *low* categories. Exposure variables, questionnaire items, response categories, and categories used in the present study are presented in Table 1.

**Table 1:** Exposure variables at baseline

**Category**	**Variables and response categories**	**Men N = 1458**		**Women N = 2705**	
		**n**	****%****	**n**	****%****
	**Computer use (CU)**				
Low	<2 hours per day	382	26	993	37
Medium	2–4 hours per day	505	35	950	35
High	>4 hours per day	564	39	748	28
	**Email/chat use**				
Low	Not at all	120	8	162	6
	<1 hour per day	906	62	1732	64
Medium	1–2 hours per day	277	19	558	21
High	>2 hours per day	151	10	236	9
	**Computer gaming**				
Low	Not at all	655	45	2050	76
	<1 hour per day	427	29	468	17
Medium	1–2 hours per day	204	14	114	4
High	>2 hours per day	168	12	61	2
	**CU without breaks**				
Low	Never	202	14	565	21
	Only occasionally	314	22	832	31
Medium	A few times per month	247	17	526	20
	A few times per week	324	22	445	17
High	Almost every day	366	25	323	12
	**CU causing lost sleep**				
Low	Never	410	28	1128	42
	Only occasionally	490	34	920	34
Medium	A few times per month	316	22	393	15
High	A few times per week	181	12	206	8
	Almost every day	57	4	46	2
	**Mobile phone use**				
Low	0–5 calls + 0–5 SMSs per day	804	55	1433	53
Medium	0–5 calls + 6–10 SMSs, or vice versa	326	22	616	23
High	>11 calls and/or >11 SMSs, or	323	22	645	24
	6–10 calls + 6–10 SMSs per day				

Frequencies and percentages in exposure variables, including categorizations into low, medium, and high, are presented. Missing values (non-responses to items) are not accounted for, which means that the n varies for the variables.

Questionnaire items: *Computer use (CU)*: On average, how much time per day have you used on a computer (total use)? *Email/chat use*: On average, how much time per day have you used on a computer for emailing and chatting (leisure/private use)? *Computer gaming*: On average, how much time per day have you spent on computer gaming (e.g., computer games or online games, private/leisure use)? *CU without breaks*: How often have you used a computer for more than 2 hours without taking a break longer than 10 minutes? *CU causing lost sleep*: How often have you lost sleep because of sitting late at night at the computer? *Mobile phone use*: How many mobile phone calls on average have you made and received per day?, and How many SMS messages on average have you sent and received per day? The time span for all items was “over the past 30 days”.

### Mental health outcome variables

Information about perceived mental health symptoms was collected from the cohort study questionnaire at baseline and follow-up. The outcome variable *currentstress* was constituted by a validated single-item stress indicator [27], with the responses divided into *yes* and *no*. The variable *sleep disturbances* was constructed for the study by including the most common sleep disturbances (difficulties falling asleep, fragmented sleep, and premature awakening) into a single item, loosely adapted from the Karolinska Sleep Questionnaire [28], with responses divided into *yes* and *no* based on clinical significance (sleep problems several times per week or more). The two depressive items from the Primary Care Evaluation of Mental Disorders (Prime-MD) screening form [29] were included in the questionnaire. It has been proposed that if one of the two items is confirmed at screening to go forward with clinical assessment of mood disorder, a procedure that has been shown to have high sensitivity for major depression diagnosis (86% [29] and 96% [30]), but lower specificity (75% [29] and 57% [30]), in primary care populations. In the youngest age group (<35 years old) in Whooley et al. [30], the sensitivity was 100% and the specificity 59%. In our cohort study group, approximately 50% of the men and almost 65% of the women confirmed at least one of the two depressive items, which indicates that the instrument is probably very sensitive but had low specificity in our study group. Therefore, we constructed two separate outcomes: *symptoms of depression (one item)* and *symptoms of depression (two items)*, the latter presumably with higher specificity. The variable *reduced performance* was based on reporting that stress, depressed mood, or tiredness had influenced performance “quite a lot” at work or school over the past 2 weeks. Mental health outcome variables, questionnaire items, response categories, and categories used in the present study are presented in Table 2.

**Table 2:** Mental health outcome variables

**Variable**	**Cohort questionnaire item**	**Response categories**	**Categories in present study**	
			**Yes**	**No**
**Current stress**	*Stress means a situation when a person feels tense, restless, nervous, or anxious or is unable to sleep at night because his/her mind is troubled all the time. Are you currently experiencing this kind of stress?*	a = *not at all*, b = *just a little*, c = *to some extent*, d = *quite a lot*, e = *very much*	d–e	a–c
**Sleep disturbances**	*How often have you had problems with your sleep these past 30 days (e.g., difficulties falling asleep, repeated awakenings, waking up too early)?*	a = *never*, b = *a few times per month*, c = *several times per week*, and d = *every day*	c–d	a–b
**Symptoms of depression** one item two items	*During the past month, have you often been bothered by:* (a) *little interest or pleasure in doing things?* (b) *feeling down, depressed, or hopeless?*	*Yes* or *No*	One item:	One item:
			(a) *Yes* or	(a) *No* and
			(b) *Yes*	(b) *No*
			Two items:	Two items:
			(a) *Yes* and	(a) *No* and
			(b) *Yes*	(b) *No*
**Reduced performance**	*Have the following complaints influenced your performance at work or in studies over the past 14 days?* (a) *stress/depressed mood?* (b) *tiredness*	a = *No*, b = *Yes, negligibly*, c = *Yes, a little*, and d = *Yes, quite a lot*	(a) d or	(a) a–c and
			(b) d	(b) a–c

### Social support

The variable social support was based on the item: *When I have problems in my private life I have access to support and help*, constructed for the study as a single item adaptation of the social support scale in the Karasek-Theorell Job Content Questionnaire [31], here relating to private life (rather than work life). Response categories were: a = *applies very poorly*; b = *appliesrather poorly*; c =* applies rather well*; d =* applies very well*. The responses were categorized as *low* (response categories a–b), *medium* (response category c), and *high* (response category d).

### Sociodemographic variables

In the multivariate analysis, potentially confounding sociodemographic factors were collected from the baseline questionnaire and adjusted for, including *relationship status*: single or in a relationship; highest completed *educational level*: elementary school (basic schooling for 6–16-year-olds), upper secondary school, or college or university studies; and *occupation*: working, studying, or other (other included being on long-term sick leave, or on parental or other leave, or being unemployed).

### Analysis

Spearman correlation analysis was used to examine associations between the different computer exposure variables, and between the exposure variables and social support. For analysis of dropout between the initial cohort baseline and 1-year follow-up, Wilcoxon’s (two-sided) two-sample test was used. The Cox proportional hazard model, using SAS PROC PHREG (SAS Institute, Inc., Cary, NC, USA), with time set to 1, was used to calculate prevalence ratios (PRs) with a 95% confidence interval (CI) for multivariate analysis of prospective associations between exposure variables high, medium, and low (reference level) and mental health outcomes (yes or no). The robust variance option (COVS) was used to produce accurate CIs [32,33].

In all prospective analyses, participants who reported symptoms at baseline were excluded from the analysis of the mental health outcome variable concerned. For example, when analyzing sleep disturbances, subjects with sleep disturbances at baseline were excluded. It is possible that the included subjects had one or more of the other mental health symptoms at baseline; however, these were not accounted for in the analysis of sleep disturbances. 

Two sets of extra analyses were performed; one, including also those with symptoms at baseline, adding the baseline value of the outcome variable as a confounder, and the other, including only those who reported symptoms at baseline.

All analyses were performed separately for the men and women. The PRs were adjusted for sociodemographic factors including relationship status, educational level, and occupation. These potential confounders were chosen based on theoretical hypothesis and were added to all analyses if p ≤ 0.10 in at least some of the analyses. Age was not considered a confounder because of the limited age span of the study group. Missing values (i.e., non-responses to items in the questionnaire) were excluded from the analyses, which means that the n varied in all analyses. PRs with a 95% CI not including 1.00 (before rounding) were considered statistically significant.

Finally, some computer exposure variables were analyzed for possible interaction with mobile phone use, by using all nine possible category combinations of the two variables in the analysis. Multivariate analyses were performed to calculate PRs using the Cox procedure, as described earlier, using the “*low–low*” category combination as reference.

All analyses were performed using the statistical software package SAS, version 9.2 (SAS Institute, Inc., Cary, NC, USA).

## Results

### Exposure, mental health symptoms, and social support in study group at baseline

Almost 40% of the men and 30% of the women were categorized as having high computer use (>4 hours per day) (Table 1). The majority spent less than 1 hour per day on leisure time email/chat use, while about 20% spent 1–2 hours per day, and 10% spent more than 2 hours per day on this activity. The percentage of men who spent >1 hour on computer gaming (*medium* or *high gaming*) was more than four times that of women. Lost sleep because of late night computer use (*CU causing lost sleep*) was more common among men than among women (Table 1). There were moderate positive associations between the exposure variables at baseline (Spearman correlation coefficients, Table 3), with the strongest associations found between *computer use* (hours per day) and frequency of *CU without breaks*. There were little if any correlations between computer exposure variables and *mobile phone use*.

**Table 3:** Associations between exposure variables at baseline

	**Email/chat use**	**Computer gaming**	**CU without breaks**	**CU causing lost sleep**	**Mobile phone use**
	**Men/Women**	**Men/Women**	**Men/Women**	**Men/Women**	**Men/Women**
**Computer use**	0.39/0.40	0.37/0.18	0.59/0.56	0.39/0.31	−0.09/0.05
**Email/chat use**		0.17/0.04	0.31/0.32	0.29/0.31	0.08/0.18
**Computer gaming**			0.36/0.17	0.27/0.19	−0.09/0.02 ns
**CU without breaks**				0.40/0.34	−0.067/0.04
**CU causing lost sleep**					−0.02 ns/0.07

Spearman correlation coefficients for the men (n = 1458) and women (n = 2705) included in the study. All correlations are statistically significant (p < 0.05) except where indicated as non-significant (ns).

The women reported current stress almost twice as often as the men (29% compared to 16%) at baseline. Twenty-three percent of the men and 34% of the women indicated sleep disturbances. Of the men, 27% reported one and 24% both symptoms of depression, and of the women, 30% reported one and 34% both symptoms of depression. Ten percent of the men and 20% of the women reported reduced performance due to stress, depressed mood, or tiredness. Among participants who were symptom-free at baseline (in terms of the outcome variable concerned), the prevalence of new cases at 1-year follow-up was as follows, for men and women respectively: current stress: 10% and 19%, sleep disturbances: 15% and 20%, symptoms of depression (one item): 24% and 28%, symptoms of depression (two items): 12% and 18%, and reduced performance: 7% and 14%.

Sixteen percent of the men and 13% of the women perceived *low social support* in private life. Forty-one percent of the men and 32% of the women reported *medium*, and 43% of the men and 56% of the women reported *high social support*.

### Prospective associations between computer exposure at baseline and mental health outcomes (new cases) at 1-year follow-up

Both *high* and *medium computer use* compared to *low computer use* at baseline were associated with sleep disturbances at 1-year follow-up for the men, but not for the women (Table 4). For men, *high computer use* was also associated with reduced performance (due to stress, depressed mood, or tiredness). *High email/chat use* was negatively associated with perceived stress for the men, but positively associated with reported sleep disturbances. For the women, both *high* and *medium email/chat use* were (positively) associated with stress and sleep disturbances. For the women, high email/chat use was also associated with symptoms of depression (one item) (PR 1.9, 95% CI 1.46–2.52, not presented in the Table). There were no other statistically significant associations with symptoms of depression (one item).

**Table 4:** Prospective associations between computer exposure at baseline and mental health outcomes (new cases) at 1-year follow-up

		**Current stress**				**Sleep disturbances**				**Symptoms of depression (two items)**				**Reduced performance**			
		**n**	**Cases (%)**	**PR**	**95% CI**	**n**	**Cases (%)**	**PR**	**95% CI**	**n**	**Cases (%)**	**PR**	**95% CI**	**n**	**Cases (%)**	**PR**	**95% CI**
**Computer use (CU)**																	
**Men**	High	440	43 (10)	1.0	0.60–1.55	398	70 (18)	**1.8**	**1.14–2.78**	173	31 (18)	1.1	0.63–2.04	400	35 (9)	**2.0**	**1.01–3.98**
	Medium	409	42 (10)	1.0	0.66–1.63	373	65 (17)	**1.8**	**1.17–2.79**	187	30 (16)	1.1	0.60–1.93	388	20 (5)	1.2	0.61–2.49
	Low	289	28 (10)	1.0		263	25 (10)	1.0		136	17 (12)	1.0		279	12 (4)	1.0	
**Women**	High	463	92 (20)	1.1	0.87–1.42	428	101 (24)	1.2	0.95–1.52	152	39 (26)	1.2	0.85–1.77	468	64 (14)	1.1	0.79–1.48
	Medium	617	117 (19)	1.0	0.81–1.29	566	94 (17)	0.8	0.65–1.06	241	64 (27)	1.2	0.90–1.70	573	84 (15)	1.1	0.82–1.48
	Low	655	122 (19)	1.0		600	128 (21)	1.0		246	56 (23)	1.0		586	75 (13)	1.0	
**Email/chat use**																	
**Men**	High	116	6 (5)	**0.4**	**0.18–0.95**	98	27 (28)	**1.9**	**1.29–2.77**	38	4 (11)	…	…	100	6 (6)	0.9	0.41–1.99
	Medium	215	20 (9)	0.8	0.52–1.31	197	31 (16)	1.1	0.77–1.63	86	13 (15)	0.9	0.50–1.51	210	16 (8)	1.3	0.73–2.25
	Low	807	88 (11)	1.0		740	102 (14)	1.0		373	61 (16)	1.0		757	45 (6)	1.0	
**Women**	High	136	37 (27)	**1.7**	**1.23–2.25**	118	35 (30)	**1.7**	**1.23–2.24**	26	8 (31)	1.4	0.74–2.50	129	21 (16)	1.3	0.84–1.94
	Medium	348	80 (23)	**1.4**	**1.11–1.76**	325	73 (22)	**1.3**	**1.01–1.62**	133	38 (29)	1.2	0.89–1.68	329	51 (16)	1.2	0.88–1.60
	Low	1248	212 (17)	1.0		1149	212 18)	1.0		477	111 (23)	1.0		1167	150 (13)	1.0	
**Computer gaming**																	
**Men**	High	136	13 (10)	0.9	0.51–1.53	107	20 (19)	1.2	0.73–1.80	43	9 (21)	1.1	0.58–1.98	108	5 (5)	0.7	0.28–1.67
	Medium	176	17 (10)	0.9	0.55–1.50	156	25 (16)	1.0	0.70–1.55	73	7 (10)	0.6	0.29–1.24	161	9 (6)	0.8	0.37–1.61
	Low	826	83 (10)	1.0		771	115 (15)	1.0		380	62 (16)	1.0		798	53 (7)	1.0	
**Women**	High	29	7 (24)	1.3	0.67–2.47	29	7 (24)	1.0	0.52–2.08	10	2 (20)	…	…	26	2 (8)	…	…
	Medium	76	15 (20)	1.0	0.65–1.63	59	17 (29)	1.4	0.92–2.09	25	11 (44)	**1.9**	**1.17–3.03**	64	7 (11)	0.8	0.39–1.61
	Low	1631	309 (19)	1.0		1509	299 (20)	1.0		605	146 (24)	1.0		1538	213 (14)	1.0	
**CU without breaks**																	
**Men**	High	280	25 (9)	0.9	0.58–1.51	250	42 (17)	1.4	0.92–2.09	100	16 (16)	0.8	0.46–1.42	253	20 (8)	1.9	0.98–3.61
	Medium	454	50 (11)	1.2	0.76–1.78	418	74 (18)	**1.5**	**1.05–2.16**	202	29 (14)	0.8	0.50–1.30	438	30 (7)	1.6	0.90–3.03
	Low	404	38 (9)	1.0		366	43 (12)	1.0		194	32 (16)	1.0		377	16 (4)	1.0	
**Women**	High	189	49 (26)	**1.6**	**1.24–2.18**	190	55 (29)	**1.7**	**1.31–2.23**	62	21 (34)	**1.8**	**1.20–2.63**	180	24 (13)	1.1	0.76–1.74
	Medium	588	121 (21)	**1.2**	**1.01–1.54**	558	120 (22)	**1.3**	**1.06–1.63**	208	62 (30)	**1.6**	**1.16–2.09**	586	94 (16)	**1.3**	**1.01–1.69**
	Low	958	161 (17)	1.0		846	147 (17)	1.0		369	76 (21)	1.0		863	105 (12)	1.0	
**CU causing lost sleep**																	
**Men**	High	174	27 (16)	**1.8**	**1.17–2.70**	146	38 (26)	**2.0**	**1.42–2.87**	53	9 (17)	1.0	0.54–2.02	155	14 (9)	**2.1**	**1.15–3.98**
	Medium	243	25 (10)	1.2	0.77–1.86	230	39 (17)	1.4	0.96–1.94	104	20 (19)	1.3	0.78–2.05	239	25 (10)	**2.5**	**1.44–4.24**
	Low	722	62 (9)	1.0		659	83 (13)	1.0		340	49 (14)	1.0		674	28 (4)	1.0	
**Women**	High	123	35 (28)	**1.7**	**1.27–2.32**	106	29 (27)	**1.5**	**1.07–2.08**	32	10 (31)	1.5	0.85–2.50	108	19 (18)	1.4	0.92–2.20
	Medium	214	55 (26)	**1.5**	**1.17–1.94**	221	55 (25)	**1.4**	**1.06–1.78**	78	31 (40)	**1.9**	**1.38–2.62**	212	41 (19)	**1.6**	**1.14–2.13**
	Low	1400	242 (17)	1.0		1270	239 (19)	1.0		529	118 (22)	1.0		1308	163 (12)	1.0	

Participants who reported symptoms at baseline were excluded from analysis of the mental health outcome concerned. The study group *n* was: for *Current stress*: 1224 men and 1915 women; for *Sleep disturbances*: 1109 men and 1764 women; for *Symptoms of depression (one item)*: 617 men and 791 women, for *Symptoms of depression (two items)*: 535 men and 693 women; and for *Reduced performance*: 1145 men and 1802 women. Missing values (non-responses to items) were excluded from the analyses, which means that the *n* varied further in the analyses. The prevalence of mental health symptoms (cases and %) at 1-year follow-up in each exposure category is shown. The prevalence ratios (PRs) with 95 % confidence intervals (CIs) were adjusted for relationship status, educational level, and occupation. PRs with a CI not including 1.00 (before rounding) are given in bold. Results of analyses with fewer than five cases are not presented, indicated as “…”.

The only clear association concerning *computer gaming* and mental health outcomes was for women, where *medium gaming* was associated with symptoms of depression (two items). Furthermore, both *high* and *medium CU without breaks* were associated with perceived stress, sleep disturbances, and symptoms of depression (two items) for the women. For the men, *medium CU without breaks* was associated with sleep disturbances. High (and, for women, *medium*) *CU causing lost sleep* was associated with stress and sleep disturbances for both the men and the women. Furthermore, *high* and *medium CU causing lost sleep* were associated with reduced performance among the men, while *medium* (but not *high*) *CU causing lost sleep* was associated with symptoms of depression (two items) and reduced performance for the women.

The extra analyses including also participants with symptoms at baseline, and adjusting for baseline health, gave results in the same direction as the results presented above. The PRs were approximately in between the PRs in Table 4 and those that resulted when including only participants with symptoms at baseline (PRs mostly around 1.0).

### Interaction effects: computer and mobile phone use

Because *computer use* and *email/chat use* were risk factors for sleep disturbances among the men, as *mobile phone use* had been in a previous study (PR 1.8, CI 1.21–2.69) [24], we tested these two exposure variables in combination with *mobile phone use*, with sleep disturbances as the outcome. Furthermore, since *CU without breaks* was a risk factor for symptoms of depression among the women, as *mobile phone use* had been in the previous study (PR 1.5, CI 1.02–2.24), these were tested in combination, with symptoms of depression (two items) as the outcome.

There seemed to be an interaction between *computer use* and *mobile phone use* in relation to sleep disturbances in a near additive fashion for the men, while for the women, only the “*high–high*” category was a risk exposure (Table 5). Men with *high email/chat use* in combination with* medium* or high *mobile phone use* had an almost tripled risk for sleep disturbances at follow-up, using “*low–low*” users as reference (Table 5). In the same analysis, for women, only effects of the *email/chat use* variable could be seen. Finally, there was a tendency towards interaction between *CU without breaks *and *mobile phone use* with symptoms of depression (two items) as outcome for the women (Table 6). Caution in interpretation is necessary since in all the combined analyses, the CIs overlapped.

**Table 5:** Interaction between computer exposure variables and mobile phone use at baseline, and sleep disturbances (new cases) at 1-year follow-up

	**MEN**						**WOMEN**					
	**Mobile phone use**						**Mobile phone use**					
	**Low**		**Medium**		**High**		**Low**		**Medium**		**High**	
Computer exposure	Cases (%)	PR (95% CI)	Cases (%)	PR (95% CI)	Cases (%)	PR (95% CI)	Cases (%)	PR (95% CI)	Cases (%)	PR (95% CI)	Cases (%)	PR (95% CI)
**Computer use**												
High	38 (16)	**2.2 (1.03–4.55)**	17 (19)	**2.9 (1.32–6.34)**	14 (22)	**3.4 (1.55–7.63)**	47 (21)	1.2 (0.84–1.66)	18 (19)	1.1 (0.69–1.76)	36 (34)	**1.8 (1.28–2.51)**
Medium	32 (14)	2.1 (1.00–4.32)	16 (21)	**3.2 (1.47–7.16)**	17 (24)	**3.9 (1.81–8.44)**	57 (17)	1.0 (0.70–1.34)	19 (15)	0.8 (0.52–1.33)	17 (15)	0.8 (0.50–1.29)
Low	8 (6)	1.0 (ref)	8 (10)	1.7 (0.69–4.38)	9 (15)	**2.4 (1.00–5.90)**	68 (19)	1.0 (ref)	36 (25)	1.3 (0.94–1.92)	24 (23)	1.1 (0.75–1.72)
**Email/chat use**												
High	11 (23)	1.8 (0.96–3.31)	9 (32)	**2.9 (1.66–5.24)**	7 (32)	**2.9 (1.47–5.59)**	14 (29)	**1.7 (1.06–2.68)**	7 (28)	1.7 (0.88–3.08)	31 (31)	**1.7 (1.08–2.68)**
Medium	18 (16)	1.4 (0.82–2.25)	9 (20)	1.8 (0.96–3.46)	4 (10)	…	30 (19)	1.1 (0.78–1.61)	19 (24)	1.4 (0.92–2.12)	23 (26)	1.5 (0.99–2.13)
Low	49 (11)	1.0 (ref)	23 (14)	1.3 (0.82–2.08)	29 (22)	**2.2 (1.41–3.34)**	126 (18)	1.0 (ref)	47 (18)	1.0 (0.75–1.37)	39 (21)	1.1 (0.78–1.47)

Participants who reported symptoms at baseline were excluded from analysis of the mental health outcome concerned. Prevalence of mental health symptoms (cases and %) at 1-year follow-up in each exposure category is shown. The prevalence ratios (PRs) with 95 % confidence intervals (CIs) were adjusted for relationship status, educational level, and occupation. PRs with a CI not including 1.00 (before rounding) are given in bold. Results of analyses with fewer than five cases are not presented, indicated as “…”.

**Table 6:** Interaction between computer exposure variables and mobile phone use at baseline, and symptoms of depression: two items (new cases), at 1-year follow-up

	**MEN**						**WOMEN**					
	**Mobile phone use**						**Mobile phone use**					
	**Low**		**Medium**		**High**		**Low**		**Medium**		**High**	
Computer exposure	Cases (%)	PR (95% CI)	Cases (%)	PR (95% CI)	Cases (%)	PR (95% CI)	Cases (%)	PR (95% CI)	Cases (%)	PR (95% CI)	Cases (%)	PR (95% CI)
*CU without breaks*												
High	12 (17)	0.9 (0.46–1.94)	4 (22)	…	0 (0)	…	10 (29)	**1.9 (1.06–3.40)**	5 (38)	**2.4 (1.13–5.09)**	6 (40)	**2.3 (1.18–4.58)**
Medium	18 (14)	1.0 (0.51–1.81)	7 (16)	1.2 (0.52–2.62)	3 (9)	…	33 (27)	**1.7 (1.09–2.52)**	14 (28)	**1.8 (1.04–3.06)**	14 (39)	**2.3 (1.38–3.87)**
Low	15 (14)	1.0 (ref)	9 (21)	1.8 (0.85–3.75)	8 (18)	1.7 (0.76–3.68)	37 (17)	1.0 (ref)	19 (22)	1.3 (0.78–2.08)	20 (29)	**1.6 (1.02–2.62)**

Participants who reported symptoms at baseline were excluded from analysis of the mental health outcome concerned. Prevalence of mental health symptoms (cases and %) at 1-year follow-up in each exposure category is shown. The prevalence ratios (PRs) with 95 % confidence intervals (CIs) were adjusted for relationship status, educational level, and occupation. PRs with a CI not including 1.00 (before rounding) are given in bold. Results of analyses with fewer than five cases are not presented, indicated as “…”.

### Social support

There were no associations between *social support* and computer exposure variables at baseline for the men, except for a very low negative association with *CU causing lost sleep* (−0.10; p < 0.001). For the women, there were very low negative associations with all computer variables (between −0.06 and −0.12; p < 0.05).

### Dropout analysis

The non-respondents at the initial cohort baseline were more often male (a difference of 17 percentage points) and were somewhat younger (an age difference of <0.1 years), more often married (a difference of 1.4 percentage points), and more often foreign-born (8 percentage points difference), compared to the study population invited to participate [26].

Furthermore, the dropout group (n = 2962) from the initial cohort baseline (n = 7125) to 1-year follow-up also had a higher proportion of men, resulting in almost twice as many women as men (65% vs. 35%) in the final study group (n = 4163). The participants in the study group had a slightly higher educational level and differed in occupation in that they were less often working and more often studying at baseline compared to the dropout group (differences of up to 10 percentage units). The level of *computer use* and *CU without breaks* was slightly higher in the study group, while *mobile phone use* was lower (differences of up to 10 percentage units). The women in the study group were less often single (34% compared to 37%) and reported a slightly higher level of *social support* (differences of up to 3 percentage units). With the exception of a lower prevalence of sleep disturbances among the men in the study group (23% compared to 27%) the prevalence of mental health symptoms at baseline was about the same among the dropouts and those who remained in the study.

## Discussion

We found prospective associations between aspects of computer use and several mental health outcomes in this population-based sample of young adults. Some of the associations were enhanced in interaction with mobile phone use. A major strength of the study is its longitudinal design, with exposure assessed among symptom-free participants prior to outcome assessment. (Extra analyses, including also participants with symptoms at baseline and adjusting for baseline health, gave results in the same direction as the presented results, although with lower PRs). However, the latency period in our study is fairly long (1 year) and we have no information about the exposure and mental health outcomes in the latency period. The mental health symptoms are common in the population and may come and go in the latency period. Hence, the time span between measurements may not be optimal for assessing possible effects of the exposure on mental health. For example, it is possible that long-term exposure is needed to develop, say, depression, or that a short-term exposure/short latency period is relevant for perceived stress.

The results were not consistent for all outcomes or for both men and women. For example, for men, hours spent on general computer use was a risk factor; by contrast, for women, it was the intensity of use, i.e., using the computer without breaks. The mental health outcome that seemed most affected for men was sleep disturbances, while for women, a more general pattern occurred.

Effects of computer exposure on perceived current stress at follow-up were mostly seen in parallel with sleep disturbances. This could be expected since the current stress item actually encompasses sleep problems. We have not assessed the relationship between the different mental health outcomes or pursued possible comorbidity within the study. For example, we do not know if other mental health symptoms were present among those participants who were symptom-free in the outcome concerned in the specific analysis. Sleep disturbances can be a first step towards depression (and is a diagnostic criterion for depression, according to the Diagnostic and Statistical Manual of Mental Disorders, 4th edition (DSM IV)). For example, in one study of young adults [34], sleep disorders was a predictor of major depression onset with an odds ratio of nearly 4. It is possible that those who developed symptoms of depression in our study already had sleep disturbances at baseline.

With regard to inconsistency of results, there was even a negative association between *high email/chat use* and stress among the men (while the association was positive for the women), implying that men who communicated via the computer more than 2 hours per day had less than half the risk to perceive stress a year later, compared to low users. On the other hand, at the same time, they had an almost double risk for developing sleep disturbances. There is of course a risk of chance findings when performing multiple tests, as in this study. However, the variables we tested were not chosen at random, and the results are in part supported by a previous study in a university sample of young adults [10], where, e.g., high chat use and high email use (as separate variables) were associated with symptoms of depression in women.

It has been claimed that women, more than men, use the computer for communication purposes [35]. In our study, men and women reported almost the exact same levels of *email/chat use* It is possible to develop and maintain large social networks via email, SMS, Facebook, web forums, and other social aspects of ICT. The positive effects of the social aspects of ICT use are often emphasized (e.g., among the subjects participants in [11]). Social support is a well-known factor that both promotes health and buffers negative effects of psychosocial strain [36,37]. Interestingly, there seemed to be little or no association between hours spent emailing or chatting and perceived social support in our study. It should be noted that the study was carried out before Facebook (and other networking services) became a widespread and popularly used application. In future studies, besides including social media use, it should be taken into consideration that social media as well as emails are increasingly accessed via the mobile phone and other portable devices, and developments seem to be towards an ever-increasing accessibility.

The *email/chat use* variable in our study concerned leisure use. Email use in work life can also induce stress. In a study among employees in an engineering company [38], time spent emailing was associated with feeling overloaded, and the overload was independent of the hours worked. While participants spent time on other activities their emails accumulated, and email use also implied interference between work and private life. It was concluded that emailing became a symbol of stress, as it seemed to be experienced as stressful regardless of the amount of work it generated, and even made the participants overlook other aspects of work that contributed to overload [38].

Social isolation is another possible consequence of high computer use, and a negative loop has been suggested [11,23]. Morahan-Martin and Schumacher [23] found support for the position that loneliness leads to increased Internet use, while Caplan [39] concludes that social anxiety (rather than loneliness) explains the preference for online social interaction, because of benefits in comparison to face-to-face communication. Such benefits, according to the author, include having control of self-presentation, phrasing, and the speed of interaction, and therefore feeling safer and more confidence than during interactions “in real life.”

Addiction to the Internet has been suggested as a possible mental health risk factor, but was not assessed in the present study. In a Norwegian cross-sectional adult population sample [17], the prevalence of Internet addiction was 1% and that of at risk users was approximately 5%. Problematic Internet use (addicted and at risk users) was more common among men and younger age groups, and was also associated with self-reported sleeping disorders and depression.

Computer gaming was more common among the men than the women in our study (as in other studies (e.g., [12]). Only a few percent of the women played games more than 2 hours per day, and playing 1–2 hours per day almost doubled the risk of symptoms of depression among the women. This was, however, the only risk category in the analysis of *computer gaming*. Computer gaming may, on the other hand, be a way of coping with stress. In a study of online game players [40], playing computer games in the workplace was associated with recovery experience. By contrast, playing long hours and at night may have detrimental effects. In a population of Internet game players, habitual gaming at night was related to an increase in depression scores in adolescents (13–17 years) and emerging adults (18–22 years), though not in the group defined as young adults (23–30 years) [41]. The association with depression was independent of total time spent playing, and gaming at night was not related to sleep problems. However, the study was cross-sectional, which limits interpretation of causality. In a laboratory study [42], computer gaming before going to bed increased sleep latency and heart rate, and decreased subjective sleepiness and rapid eye movement (REM) sleep, compared to controlled conditions. Getting stuck behind the screen and thus losing sleep was reported as a problem in our interview study [11] and was the basis for constructing the variable *CU causing lost sleep*, which also turned out to be a risk factor for several mental health outcomes including reduced performance, for both men and women. It may be argued that the variable involves circular reasoning, since it encompasses lost sleep as part of the exposure, which at the same time may be considered an outcome.

High ICT use can mirror a hectic lifestyle with high demands (extrinsic as well as intrinsic). It can mirror a lifestyle with lack of recovery, and have potential effects on sleep. Moreover, it is possible that ICT use contributes to a sedentary lifestyle. A sedentary lifestyle can have negative effects on mental health, while physical activity has positive effects on mental health and is acknowledged as a possible complementary treatment for depression and stress-related disorders [43,44]. A review suggests that sedentary behavior, including TV viewing and computer/Internet use, is associated with an increased risk of depression [45]. However, the possible role of physical activity was not assessed in the present study.

### Methodological considerations

There are several limitations in the study, which need to be considered, for example the inconsistencies in the results, as discussed above. The study was performed in a population-based sample of young adults, which is one of its strengths. However, caution must be taken when generalizing the results. The study suffered from a high dropout rate, as is common in questionnaire studies administered in the general population (and probably more so among young adults), resulting in, e.g., women and native-born Swedes being overrepresented in the study group. It is possible that ICT exposure in the study group differs from that of the source population. There is probably a healthy participation selection bias in the cohort, which may be further enhanced when excluding participants with symptoms at baseline before analysis, as the remaining participants might differ from the source population in several aspects including health and resilience. We did adjust for potential confounding by occupation, educational level, and relationship status. However, this was done only at baseline and situations may have changed during the latency period, which will not have been accounted for. Furthermore, we adjusted for potential confounding in all analyses, even though all confounders were not relevant in all analyses. Moreover, geographical confounding may be present because half of the source population was from one region of Sweden and the other half was from the rest of the country. Gender bias was eliminated by separately analyzing men and women.

There are several limitations in using a questionnaire to collect information on exposure and health aspects. Recall bias and recall difficulties are most likely present. Agreement between self-reported and registered exposure clearly was low in one validity study concerning computer use [46] where more than 80% of respondents misclassified their computer use, with almost all respondents overestimating their use. Perhaps in a future study, we would want to use objective exposure assessment via technical registration, rather than self-report.

Some of the mental health outcomes used in the study were not validated (sleep disturbances and reduced performance due to stress, depressed mood or tiredness), including the social support variable, which is a limitation of the study. It is important to point out that the study concerns subjective symptom reports and not actual mental disorders or diagnoses. The prevalence of reported depressive symptoms was alarmingly high in our study group; approximately 50% of the men and almost 65% of the women confirmed at least one of the two depressive items. Following the suggested PRIME-MD procedure [29,30], a predictive power of 33% [30] would imply that about 20% of the study group was clinically depressed. However, the prevalence of depression in a population such as the one included in this study is most likely lower than in primary care populations. For comparison, the 1-month prevalence of depression among Finnish young adults (20–24 years of age) was 9.6% [47]. Suspecting that the instrument would be too sensitive for our population, we chose to adapt the analysis accordingly, expecting that the two-item outcome would have higher specificity than if following the suggested procedure.

The computer use variables based on time spent at the computer in our study did not permit us to evaluate extreme use, since the cutoff for the highest category of general computer use was 4 hours per day, which was the time that almost 40% of the men and 30% of the women spent on the computer. The cutoff for the *high* category in *email/chat use* and *computer games* was 2 hours per day. It is possible that more extreme exposure is more hazardous to health than the tested exposures and having more categories with higher cutoffs may have enabled a more detailed dose–response analysis. Perhaps the variables concerning intensity of use (i.e., *CU without breaks*, and *CU causing lost sleep*) can reflect a more extreme and, possibly, destructive use, than those concerning mere hours of use. Also, it is important, though difficult, to keep up to date with advances in technology and applications, and to create items that sufficiently capture relevant exposure, especially in longitudinal studies.

### Implications

Aspects of ICT use can contain risk factors for mental wellbeing among young adults, and may be markers for (other) mechanisms associated with mental health risks. Consequently, it seems desirable to support healthy use of modern technologies in order to prevent possible destructive uses or effects, since ICT is an ever-increasing part of daily life, at work, at school, and at leisure. Since sleep and recovery are essential for maintained health, further studies could focus on mechanisms relating ICT use to sleep disturbances, to deepen our understanding and develop meaningful and evidence-based intervention programs.

## Conclusions

Time spent on general computer use was prospectively associated with sleep disturbances and reduced performance for the men included in this study. For the women, using the computer without breaks was a risk factor for several mental health outcomes. Some associations were enhanced in interaction with mobile phone use. Using the computer at night and thus losing sleep was associated with most mental health outcomes for both men and women. Further studies should focus on mechanisms relating ICT use to sleep disturbances.

## Competing interests

The authors declare that they have no competing interests.

## Authors' contributions

ST, AH, and MH designed the study. ST performed the data analysis and wrote the manuscript. AH and MH supervised the data analysis, and discussed and contributed to the manuscript. All authors have read and approved the final manuscript.

## Pre-publication history

The pre-publication history for this paper can be accessed here:

http://www.biomedcentral.com/1471-244X/12/176/prepub

## References

[B1] Gerr F Monteilh CP Marcus M Keyboard use and musculoskeletal outcomes among computer users J Occup Rehabil 2006 16 3 265 277 16802184 10.1007/s10926-006-9037-0

[B2] Gustafsson E Hagberg M Computer mouse use in two different hand positions: Exposure, comfort, exertion and productivity Appl Ergon 2003 34 2 107 113 10.1016/S0003-6870(03)00005-X 12628567

[B3] Ekman A Andersson A Hagberg M Hjelm EW Gender differences in musculoskeletal health of computer and mouse users in the Swedish workforce Occup Med 2000 50 8 608 613 10.1093/occmed/50.8.608 11220032

[B4] Nakazawa T Okubo Y Suwazono Y Kobayashi E Komine S Kato N Nogawa K Association between duration of daily VDT use and subjective symptoms Am J Ind Med 2002 42 5 421 426 10.1002/ajim.10133 12382255

[B5] Berg M Arnetz BB Liden S Eneroth P Kallner A Techno-stress. A psychophysiological study of employees with VDU-associated skin complaints J Occup Med 1992 34 7 698 701 1386626

[B6] Arnetz BB Wiholm C Technological stress: Psychophysiological symptoms in modern offices J Psychosom Res 1997 43 1 35 42 10.1016/S0022-3999(97)00083-4 9263929

[B7] Johansson-Hidén B Wästlund E Wallin S Reflecting on ICT and stress: conceptional connections and a suggested application HumanIT 2003 Karlstad: Karlstad University Studies

[B8] Statistics Sweden Use of computers and the Internet by private persons in 2010 [In Swedish and parts in English] 2011 http://www.scb.se

[B9] Nordicom: Internetbarometer 2010 [In Swedish] 2011 Sweden: University of Gothenburg http://www.nordicom.gu.se

[B10] Thomée S Eklöf M Gustafsson E Nilsson R Hagberg M Prevalence of perceived stress, symptoms of depression and sleep disturbances in relation to information and communication technology (ICT) use among young adults - an explorative prospective study Comput Human Behav 2007 23 3 1300 1321 10.1016/j.chb.2004.12.007

[B11] Thomée S Dellve L Härenstam A Hagberg M Perceived connections between information and communication technology use and mental symptoms among young adults - a qualitative study BMC Publ Health 2010 10 1 66 10.1186/1471-2458-10-66 PMC2836296 20152023

[B12] Punamäki RL Wallenius M Nygard CH Saarni L Rimpela A Use of information and communication technology (ICT) and perceived health in adolescence: the role of sleeping habits and waking-time tiredness J Adolesc 2007 30 4 569 585 10.1016/j.adolescence.2006.07.004 16979753

[B13] Carbonell X Guardiola E Beranuy M Bellés A A bibliometric analysis of the scientific literature on Internet, video games, and cell phone addiction J Med Libr Assoc 2009 97 2 102 107 10.3163/1536-5050.97.2.006 19404500 PMC2670219

[B14] Young KS Internet addiction: the emergence of a new clinical disorder Cyberpsychol Behav 1998 1 3 237 244 10.1089/cpb.1998.1.237

[B15] Shapira NA Goldsmith TD Keck PE Jr Khosla UM McElroy SL Psychiatric features of individuals with problematic internet use J Affect Disord 2000 57 1–3 267 272 10708842 10.1016/s0165-0327(99)00107-x

[B16] Yellowlees PM Marks S Problematic Internet use or Internet addiction? Comput Hum Behav 2007 23 3 1447 1453 10.1016/j.chb.2005.05.004

[B17] Bakken IJ Wenzel HG Gotestam KG Johansson A Oren A Internet addiction among Norwegian adults: a stratified probability sample study Scand J Psychol 2009 50 2 121 127 10.1111/j.1467-9450.2008.00685.x 18826420

[B18] Cheung LM Wong WS The effects of insomnia and internet addiction on depression in Hong Kong Chinese adolescents: an exploratory cross-sectional analysis J Sleep Res 2011 20 2 311 317 10.1111/j.1365-2869.2010.00883.x 20819144

[B19] Lam LT Peng ZW Effect of pathological use of the internet on adolescent mental health: a prospective study Arch Pediatr Adolesc Med 2010 164 10 901 906 10.1001/archpediatrics.2010.159 20679157

[B20] Mentzoni RA Brunborg GS Molde H Myrseth H Skouverøe KJM Hetland J Pallesen S Problematic video game use: estimated prevalence and associations with mental and physical health Cyberpsychol Behav Soc Netw 2011 14 10 591 596 10.1089/cyber.2010.0260 21342010

[B21] Chou C Tsai MJ Gender differences in Taiwan high school students' computer game playing Comput Hum Behav 2007 23 1 812 824 10.1016/j.chb.2004.11.011

[B22] Gentile DA Choo H Liau A Sim T Li D Fung D Khoo A Pathological video game use among youths: a two-year longitudinal study Pediatrics 2011 127 2 e319 e329 10.1542/peds.2010-1353 21242221

[B23] Morahan-Martin J Schumacher P Loneliness and social uses of the Internet Comput Hum Behav 2003 19 6 659 671 10.1016/S0747-5632(03)00040-2

[B24] Thomée S Härenstam A Hagberg M Mobile phone use and stress, sleep disturbances, and symptoms of depression among young adults - a prospective cohort study BMC Publ Health 2011 11 1 66 10.1186/1471-2458-11-66 PMC3042390 21281471

[B25] The United Nations Youth and the United Nationshttp://www.un.org/esa/socdev/unyin/qanda.htm

[B26] Ekman A Ahlstrand C Andrén M Boström M Dellve L Eriksson J Gustafsson E Hagberg J Lindegård A Thomée S Ung Vuxen - Basenkät (Young adults - baseline questionnaire) [In Swedish]. In: Rapport från Arbets- och miljömedicin [Occupational and Environmental Report] No 118. Occupational and Environmental Medicine 2008 Gothenburg, Sweden: University of Gothenburg

[B27] Elo A Leppänen A Jahkola A Validity of a single-item measure of stress symptoms Scand J Work Environ Health 2003 29 6 441 451 10.5271/sjweh.750 14712852

[B28] Kecklund G Åkerstedt T The psychometric properties of the Karolinska Sleep Questionnaire J Sleep Res 1992 1 Suppl 1 113

[B29] Spitzer RL Williams JB Kroenke K Linzer M de Gruy FV III Hahn SR Brody D Johnson JG Utility of a new procedure for diagnosing mental disorders in primary care. The PRIME-MD 1000 study J Am Med Assoc 1994 272 22 1749 1756 10.1001/jama.1994.03520220043029 7966923

[B30] Whooley MA Avins AL Miranda J Browner WS Case-finding instruments for depression. Two questions are as good as many J Gen Intern Med 1997 12 7 439 445 10.1046/j.1525-1497.1997.00076.x 9229283 PMC1497134

[B31] Karasek R Theorell T Healthy Work 1990 New York: Basic Books

[B32] Coutinho LMS Scazufca M Menezes PR Methods for estimating prevalence ratios in cross-sectional studies Rev Saude Publica 2008 42 6 992 998 10.1590/S0034-89102008000600003 19009156

[B33] Deddens JA Petersen MR Approaches for estimating prevalence ratios Occup Environ Med 2008 65 7 501 486 481 10.1136/oem.2007.034777 18562687

[B34] Breslau N Roth T Rosenthal L Andreski P Sleep disturbance and psychiatric disorders: a longitudinal epidemiological study of young adults Biol Psychiatry 1996 39 6 411 418 10.1016/0006-3223(95)00188-3 8679786

[B35] Shaw LH Gant LM Users divided? Exploring the gender gap in Internet use Cyberpsychol Behav 2002 5 6 517 527 10.1089/109493102321018150 12556114

[B36] Stansfeld S Candy B Psychosocial work environment and mental health–a meta-analytic review Scand J Work Environ Health 2006 32 6 443 462 10.5271/sjweh.1050 17173201

[B37] Cohen S Psychosocial models of the role of social support in the etiology of physical disease Health Psychol 1988 7 3 269 297 3289916 10.1037//0278-6133.7.3.269

[B38] Barley SR Meyerson DE Grodal S E-mail as a source and symbol of stress Organ Sci 2011 22 4 887 906 10.1287/orsc.1100.0573

[B39] Caplan SE Relations among loneliness, social anxiety, and problematic internet use Cyberpsychol Behav 2007 10 2 234 242 10.1089/cpb.2006.9963 17474841

[B40] Reinecke L Games at work: the recreational use of computer games during working hours Cyberpsychol Behav 2009 12 4 461 465 10.1089/cpb.2009.0010 19619038

[B41] Lemola S Brand S Vogler N Perkinson-Gloor N Allemand M Grob A Habitual computer game playing at night is related to depressive symptoms Personal Individ Differ 2011 51 2 117 122 10.1016/j.paid.2011.03.024

[B42] Higuchi S Motohashi Y Liu Y Maeda A Effects of playing a computer game using a bright display on presleep physiological variables, sleep latency, slow wave sleep and REM sleep J Sleep Res 2005 14 3 267 273 10.1111/j.1365-2869.2005.00463.x 16120101

[B43] Sanchez-Villegas A Ara I Guilleén-Grima F Bes-Rastrollo M Varo-Cenarruzabeitia JJ Martínez-Gonzílez MA Physical activity, sedentary index, and mental disorders in the SUN cohort study Med Sci Sports Exerc 2008 40 5 827 834 10.1249/MSS.0b013e31816348b9 18408617

[B44] Jonsdottir IH Stress, exercise and consequences for memory function and affective disorders Neurol Cogn Neurosci 2006 6 1 2 6

[B45] Teychenne M Ball K Salmon J Sedentary behavior and depression among adults: a review Int J Behav Med 2010 17 4 246 254 10.1007/s12529-010-9075-z 20174982

[B46] Ijmker S Leijssen JNM Blatter BM van der Beek AJ van Mechelen W Bongers PM Test-retest reliability and validity of self-reported duration of computer use at work Scand J Work Environ Health 2008 34 2 113 119 10.5271/sjweh.1220 18470442

[B47] Aalto-Setala T Marttunen M Tuulio-Henriksson A Poikolainen K Lonnqvist J One-month prevalence of depression and other DSM-IV disorders among young adults Psychol Med 2001 31 5 791 801 11459377 10.1017/s0033291701004081

